# Food Allergy in Lebanon: Is Sesame Seed the "Middle Eastern" Peanut

**DOI:** 10.1097/WOX.0b013e318204b745

**Published:** 2011-01-15

**Authors:** Carla Irani, George Maalouly, Mirna Germanos, Hassan Kazma

**Affiliations:** 1 Hotel Dieu de France hospital, Internal Medicine Department, Allergy and Immunology Section; 2 Hotel Dieu de France hospital, Laboratory Department, Division of Hematology and Immunology; 3 Hammoud Hospital, Laboratory Department, Division of Hematology and Immunology

**Keywords:** food allergy, sesame, peanut

## Abstract

A randomly sampled, cross-sectional serology test-based survey was conducted in Lebanon to describe the pattern of food allergy among Lebanese population. The prevalence of specific Immunoglobulin E (IgE) to food allergens was investigated in 20 laboratories in different regions of Lebanon by an immunoblot assay over a 1 year period. Clinical correlation was determined in two university hospitals. There were 1842 patients with suspected IgE-mediated food allergic reactions tested for specific IgE upon their physician's request. Clinical correlation was done in 93 patients. We identified 386 out of 1842 (20.95%) patients with positive specific IgE to food allergens. The clinical presentations were cutaneous, digestive, and anaphylaxis. The major cause of allergy was cow's milk in infants and young children, hazelnut and wheat flour in adults. Although specific IgE to peanut in infants, children, and adults were higher than for sesame, peanut-induced allergic reactions were mild, in contrary to sesame where anaphylaxis was the only clinical manifestation. Recently, sesame has been recognized as an increasingly frequent and potentially severe allergen. Further studies with double-blind, placebo-controlled food challenge are needed to establish the real prevalence of food allergy in Lebanon, and to determine the most common allergens taking in consideration the nutritional habits of our population.

## Introduction

Food allergy is a common and serious allergic disease affecting both children and adults. There are no epidemiologic studies concerning the prevalence of food allergy in Lebanon. Although the gold standard for diagnosing food allergy is the double-blind, placebo-controlled food challenge, diagnostic food-specific IgE levels might assist in diagnosing food allergies, circumventing the need for risky food challenges[1] especially for nuts. The objective of this study was to estimate the most common food allergens revealed by positive specific IgE, among the Lebanese population.

## Materials and methods

In a cross-sectional study, the prevalence of positive specific Immunoglobulin E (IgE) to food allergens was investigated in 20 laboratories in different regions of Lebanon by the *AllergyScreen*-Test over a 1 year period. The *Allergy-Screen*-Test is an immunoblot assay aimed to the semi-quantitative determination of circulating allergen-specific IgE in human serum. The method used in all laboratories for IgE detection was the CAP-RAST. A panel of 20 allergens was used for the test including hazelnut, peanut, walnut, almond, milk, egg white, egg yolk, casein, potato, celery, carrot, tomato, cod fish, crab, orange, apple, wheat flour, rye flour, sesame seed, and soy bean.

There were 1842 patients of all ages, with a suggestive history of IgE-mediated food allergy, tested for specific IgE upon their physician's request. Of these, 337 (18.29%) were referred from the allergy clinic of 2 university hospitals. Clinical correlation was studied in 93 of the 337 patients (27.6%), because they were found to have positive specific IgE to food allergens. This group of patients followed clinically represents 5% of all patients originally included in the study.

## Results

**Figure 1:**
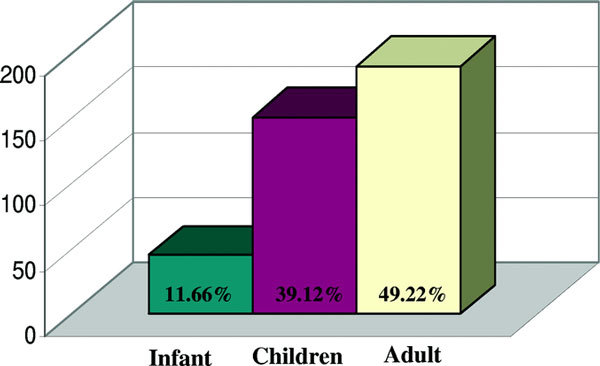
**Prevalence of food specific IgE antibodies in 386 patients**.

**Figure 2:**
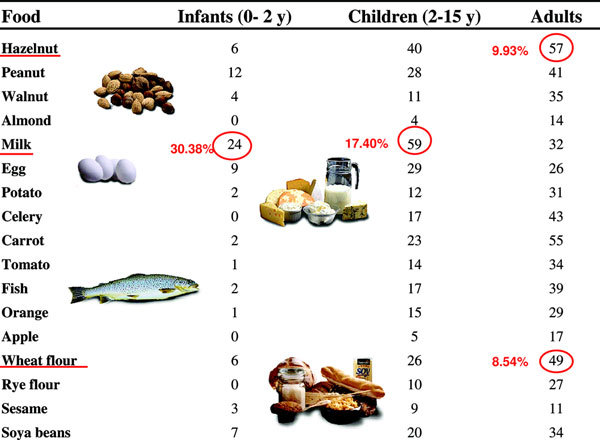
**Results**.

**Figure 3:**
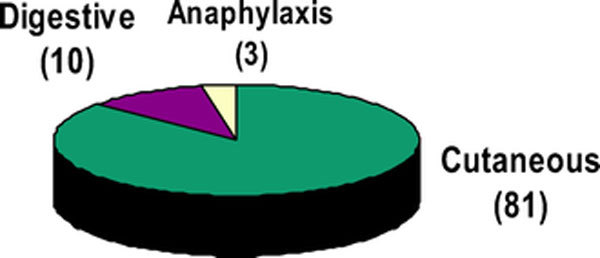
**Clinical symptoms of food reactions in 93 patients**.

We identified 386 of 1842 (20.95%) patients with positive specific IgE to food allergens; 11.66% were infants, 39.12% were children, and 40.22% were adults. Figure 1 depicts prevalence of food specific antibodies in 386 patients, Figure 2 depicts the distribution of positive IgE specific to the food tested in these patients, and Figure 3 shows the clinical presentations of food allergy in 93 patients. The most common positive IgEs were to cow's milk in infants and young children (30.38 and 17.40%, respectively), hazelnut and wheat flour in adults (9.93 and 8.54%, respectively). Positive specific IgE to sesame seed were 3.79% in infants, 2.65% in children, and 1.91% in adults, whereas positive specific IgE to peanut were higher, 15.18, 8.26, and 7.14%, respectively. There were 60% of the 93 patients who had a history of allergic rhinitis and/or asthma, and up to 80% had atopic dermatitis.

The clinical manifestation of food allergy in those patients were urticaria/angiodema (n = 81, 86.17%), followed by gastro-intestinal symptoms (n = 10, 10.63%). One of the patients had both cutaneous and digestive symptoms. Anaphylaxis was the presenting symptom in 3 of the 93 (3.19%) patients; 2 of these were sesame seed-induced cases; the third case was to lentils.

Although specific IgE to peanut in infants (15.18%), children (8.26%), or adults (7.14%) were more common than for sesame seed (3.79, 2.65, and 1.91%, respectively), among the 93 patients reviewed clinically, peanut-induced allergic reactions were mild, in contrary to sesame seed where anaphylaxis was the only clinical manifestation. All the cases of anaphylaxis to sesame seed, and allergy to peanut had later on, a prick skin test to confirm the diagnosis.

We did an open food challenge in 4 patients after 3 years of elimination, 2 of them to milk and were successful, and 2 to eggs, 1 of them showed no allergenicity, the second patient remained allergic. Of course, a double-blind, placebo-controlled food challenge is the gold standard for the diagnosis, but it was not done. As for the sesame, 1 patient showed a negative skin testing after 3 years of elimination but the mother is still refusing to do the challenge, the second case is still showing sensitization by skin test after 4 years of elimination

## Discussion

Adverse reactions to food, that is, food allergy and intolerance have gained considerable attention. Food allergy is believed to be responsible for most immediate-type food-induced hypersensitivity reactions. Clinically, these reactions are characterized by a variety of signs and symptoms that occur within minutes or hours after consumption of the offending food. Reactions may be limited or more generalized with involvement of the skin, nose, eyes, and/or lungs. In more severe cases, cardiovascular symptoms including hypotension, shock, cardiac dysrhythmias, and death can occur. In food-allergic individuals, IgE is produced against naturally occurring food components, primarily glycoproteins that usually retain their allergenicity after heating and/or proteolysis [2].

Whereas in the United States, peanuts and tree nuts (walnuts, pecans, pistachios, cashews, and others) are responsible for the majority of severe anaphylactic reactions, followed by shellfish (crustaceans and mollusks) and fish, in other parts of the world, foods that are prominent in the regional diet are leading culprits, including sesame seed in middle-east populations and seafood in China.

Sesame seed allergy is a significant, serious, and growing problem especially in the Middle Eastern region where the introduction of sesame seed is early in the diet, but in small quantities. Evidence was found for increased incidence of sesame seed allergy during the past 5 decades, with the most reports pointing to the developed countries [3]. Sesame seeds are used for flavoring many prepared foods [4] and was included among the allergens required by The European Union legislation in 2005 to be listed in food labels (in addition to milk, egg, fish, crustacean shellfish, peanuts, soybeans, tree nuts, cereals containing gluten, celery, and mustard) [5]. In 2007, the European Union added molluscan shellfish and lupine to the labeling list. Usual age of onset of sesame seed allergy is 6-36 months; only 20% of cases resolve by 7 years, the majority being persistent into adulthood [6].

Clinically, most sesame seed allergy present in at least 2 major forms: immediate hypersensitivity, often expressed as systemic anaphylaxis, associated with positive skin prick test and/or IgE antibody test results to sesame proteins with some cross-reactivity with other foods, and delayed hypersensitivity to lignin-like compounds in sesame oil clinically expressed as contact allergic dermatitis. There are a few cases in the literature of immediate hypersensitivity to sesame seed with negative skin prick test and/or IgE antibody test results that were confirmed by oral challenge tests [7]. In one study of sesame seed allergy in children, immediate reaction to a minimal amount of sesame was characteristic; skin was the most common site of involvement, followed by respiratory and gastrointestinal systems; tolerance developed in only 20% of the patients, and high sIgE (> 0.15 IU) was demonstrated only in 75% of those in which it was examined [8]. Epitopes of the 14-kd beta-globulin, the major allergen of sesame seed, were recently identified [9,10].

## Conclusion

Recently, sesame seed has been recognized as an increasingly frequent and potentially severe allergen even in westernized countries, furthermore we think awareness in Lebanon and in the Middle East regarding sesame seed allergy is very important. Because no data are available in Lebanon, we consider our study a fair start. Anaphylaxis to sesame seed has been described with negative SPT and IgE levels [11-13]. Pertinent history, along with laboratory studies, such as specific IgE, are the first step in the diagnostic process of food allergy [14]. Allergy prick skin test are usually more specific when they are done by specialized health care professionals. Further studies with double-blind, placebo-controlled food challenge are needed to establish the real prevalence of food allergy in Lebanon, and to determine the most common allergens taking in consideration the nutritional habits of our population.

## References

[B1] Sampson H Food allergy J Allergy Clin Immunol 2003 4 2 540 547 10.1067/mai.2003.134 12592300

[B2] Ebo DG Stevens WJ IgE-mediated food allergy-extensive review of the literature Acta Clin Belg 2001 4 4 234 247 11603253 10.1179/acb.2001.035

[B3] Gangur V Kelly C Navaluri L Sesame allergy: a growing food allergy of global proportions? Ann Allergy Asthma Immunol 2005 4 1 4 11 quiz 11-3, 44 10.1016/S1081-1206(10)61181-7 16095135

[B4] Altman DR Chiaramonte LT Public perception of food allergy J Allergy Clin Immunol 1996 4 6 1247 1251 10.1016/S0091-6749(96)70192-6 8648020

[B5] Taylor SL Hefle SL Food allergen labeling in the USA and Europe Curr Opin Allergy Clin Immunol 2006 4 3 186 190 10.1097/01.all.0000225158.75521.ad 16670512

[B6] Sampson H Peanut allergy N Engl J Med 2002 4 17 1295 1299 10.1056/NEJMcp012667 11973367

[B7] Dalal I Binson I Levine A Somekh E Ballin A Reifen R The pattern of sesame sensitivity among infants and children Pediatr Allergy Immunol 2003 4 4 312 316 10.1034/j.1399-3038.2003.00040.x 12911511

[B8] Cohen A Goldberg M Levy B Leshno M Katz Y Sesame food allergy and sensitization in children: the natural history and long-term follow-up Pediatr Allergy Immunol 2007 4 3 217 223 Epub 2007 Mar 7 10.1111/j.1399-3038.2006.00506.x 17346302

[B9] Wolff N Cogan U Admon A Dalal I Katz Y Allergy to sesame in humans is associated primarily with IgE antibody to a 14 kDa 2S albumin precursor Food Chem Toxicol 2003 4 8 1165 1174 10.1016/S0278-6915(03)00107-8 12842185

[B10] Wolff N Yannai S Karin N Levy Y Reifen R Dalal I Cogan U Identification and characterization of linear B-cell epitopes of beta-globulin, a major allergen of sesame seeds J Allergy Clin Immunol 2004 4 5 1151 1158 10.1016/j.jaci.2004.07.038 15536424

[B11] Leduc V Moneret-Vautrin DA Tzen JT Morisset M Guerin L Kanny G Identification of oleosins as major allergens in sesame seed allergic patients Allergy 2006 4 349 356 10.1111/j.1398-9995.2006.01013.x 16436145

[B12] Cohen A Goldberg M Levy B Leshno M Katz Y Sesame food allergy and sensitizationin children: the natural history and long-term follow-up Pediatr Allergy Immunol 2007 4 217 223 10.1111/j.1399-3038.2006.00506.x 17346302

[B13] Zavalkoff S MDCM, FRCPC letters to the editor J Allergy Clin Immunol 2008 4 1508 1510 10.1016/j.jaci.2008.04.012 18539199

[B14] Maloney JM Rudengren M Ahlstedt S Bock SA Sampson HA The use of serum-specific IgE measurements for the diagnosis of peanut, tree nut, and seed allergy J Allergy Clin Immunol 2008 4 1 145 151 Epub 2008 May 27 10.1016/j.jaci.2008.04.014 18502490

